# Supplementary material for: Adaptive Responses of the Sea Anemone Heteractis crispa to the Interaction of Acidification and Global Warming
Source: Animals (Basel). 2022 Aug 31;12(17):2259. doi: 10.3390/ani12172259 (PMC9454579; doi:10.3390/ani12172259)
Supplement: Supplementary file 1 [file animals-12-02259-s001.zip › Supplementary Materials/Supplementary Tables.pdf]

Table S1. Seawater carbonate chemistry under experimental conditions.

Treatments	pCO_2	HCO_3^- (μmol kg^{-1})	CO_3^{2-} (μmol kg^{-1})	Ω_{calc}	Ω_{arag}	TA	Salinity
Control	473.3 ± 7.5	1563.1 ± 12.5	132.6 ± 5.8	1.78 ± 0.15	1.19 ± 0.02	1603.5 ± 3.5	35.63 ± 1.4
AC	923.2 ± 17.2	1834.7 ± 7.9	77.9 ± 1.8	1.37 ± 0.03	0.77 ± 0.05	2114.8 ± 6.9	36.55 ± 1.1
WC	481.8 ± 11.5	1615.7 ± 20.7	141.3 ± 6.7	2.13 ± 0.04	1.32 ± 0.01	1698.7 ± 5.1	36.24 ± 2.1
CC	937.5 ± 13.3	1871.4 ± 14.0	85.1 ± 2.7	1.49 ± 0.08	1.13 ± 0.03	2235.3 ± 3.1	35.47 ± 1.7

AC, acidification conditions; WC, warming conditions; CC, combined conditions; Ω_{calc}, calcite saturation state; Ω_{arag}, aragonite saturation state; TA, total alkalinity.

Table S2. Changes in the levels of symbiont sterols in response to stress on day 3.

Sterol	Control	AC	Control	WC	Control	CC
Cholesterol Content (% of total sterols)	11.87 ± 0.69	10.89 ± 0.19	11.56 ± 1.06	9.3 3± 0.74*	11.73 ± 0.93	7.83 ± 0.33*
Total sterols (μg·mg^{-1} DW)	16.98 ± 0.77	16.01 ± 0.33	17.21 ± 0.47	13.71 ± 0.78*	17.03 ± 0.37	11.38 ± 0.52*

Asterisks (*) indicate significant differences compared to the control conditions (p-values < 0.05). Three biological replicates were established under each set of treatment conditions. Average values ± errors are shown. AC, acidification conditions; WC, warming conditions; CC, combined conditions; DW, dry weight.

Table S3. Changes in the levels of symbiont sterols in response to stress on day 15.

Sterol	Control	AC	Control	WC	Control	CC
Cholesterol Content (% of total sterols)	11.93 ± 0.41	9.72 ± 0.57*	11.43 ± 0.56	7.54 ± 0.44*	10.99 ± 1.13	5.33 ± 0.91*
Total sterols (μg·mg^{-1} DW)	17.25 ± 0.27	14.61 ± 0.33*	17.51 ± 0.74	10.71 ± 1.21*	16.83 ± 1.3	8.98 ± 0.72*

Asterisks (*) indicate significant differences compared with the control conditions (p-values < 0.05). Three biological replicates were established under each set of treatment conditions. Average values ± errors are shown. AC, acidification conditions; WC, warming conditions; CC, combined conditions; DW, dry weight.
