# Supplementary figures and images for: Adaptive Responses of the Sea Anemone Heteractis crispa to the Interaction of Acidification and Global Warming
Source: Animals (Basel). 2022 Aug 31;12(17):2259. doi: 10.3390/ani12172259 (PMC9454579; doi:10.3390/ani12172259)

A

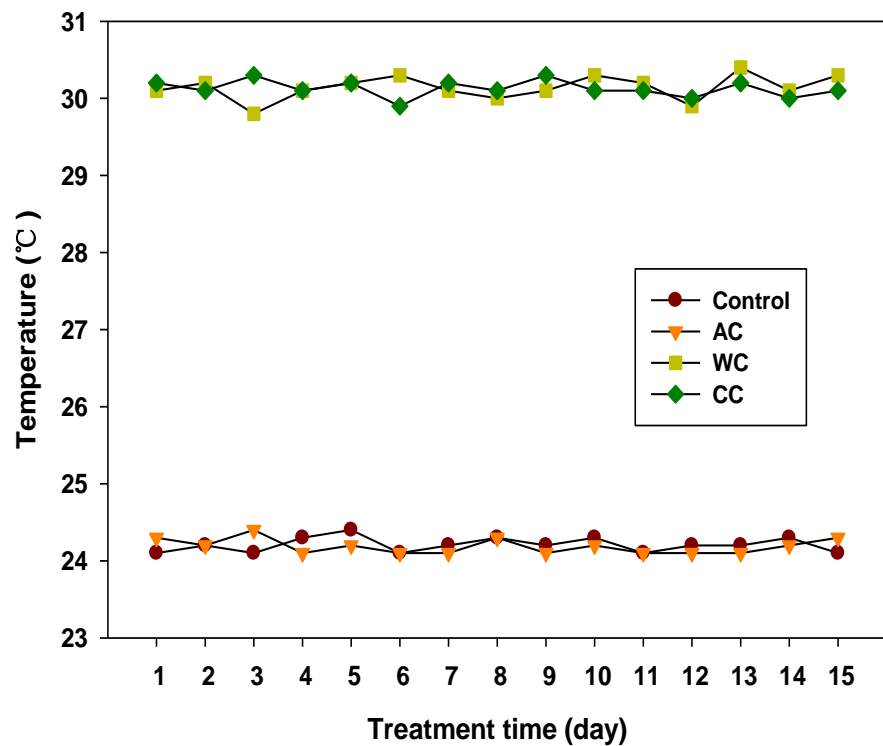

B

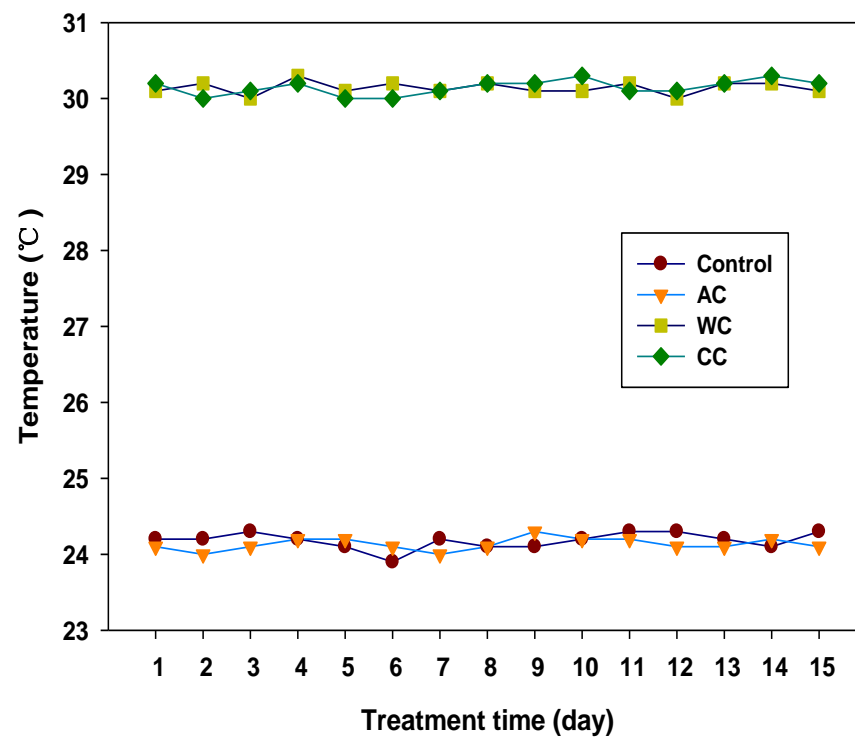

Supplement: Supplementary file 1 [file animals-12-02259-s001.zip › Supplementary Materials/Supplementary Figures/Figure S1 .pdf]

A

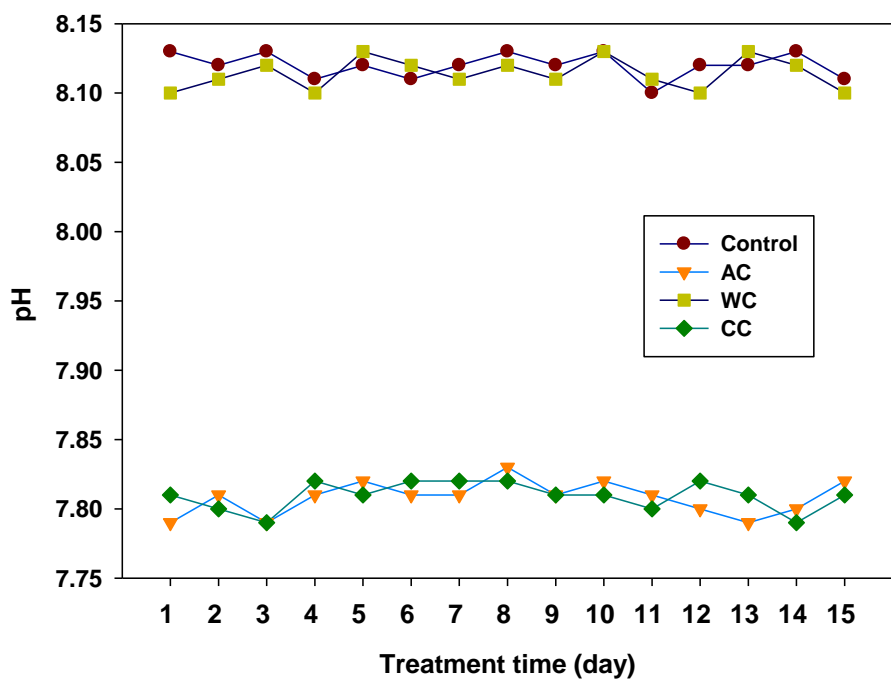

B

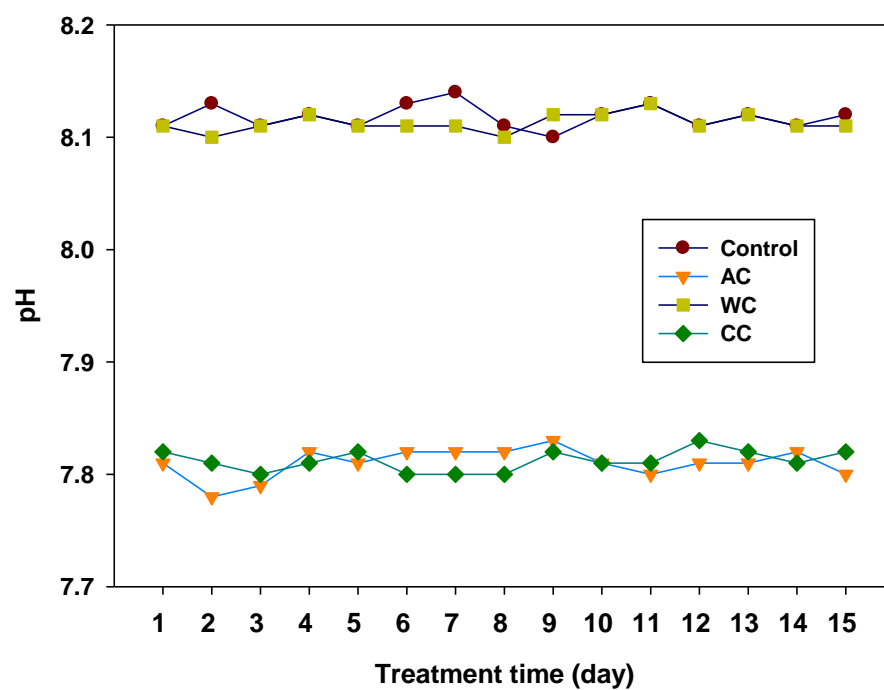

Supplement: Supplementary file 1 [file animals-12-02259-s001.zip › Supplementary Materials/Supplementary Figures/Figure S2.pdf]

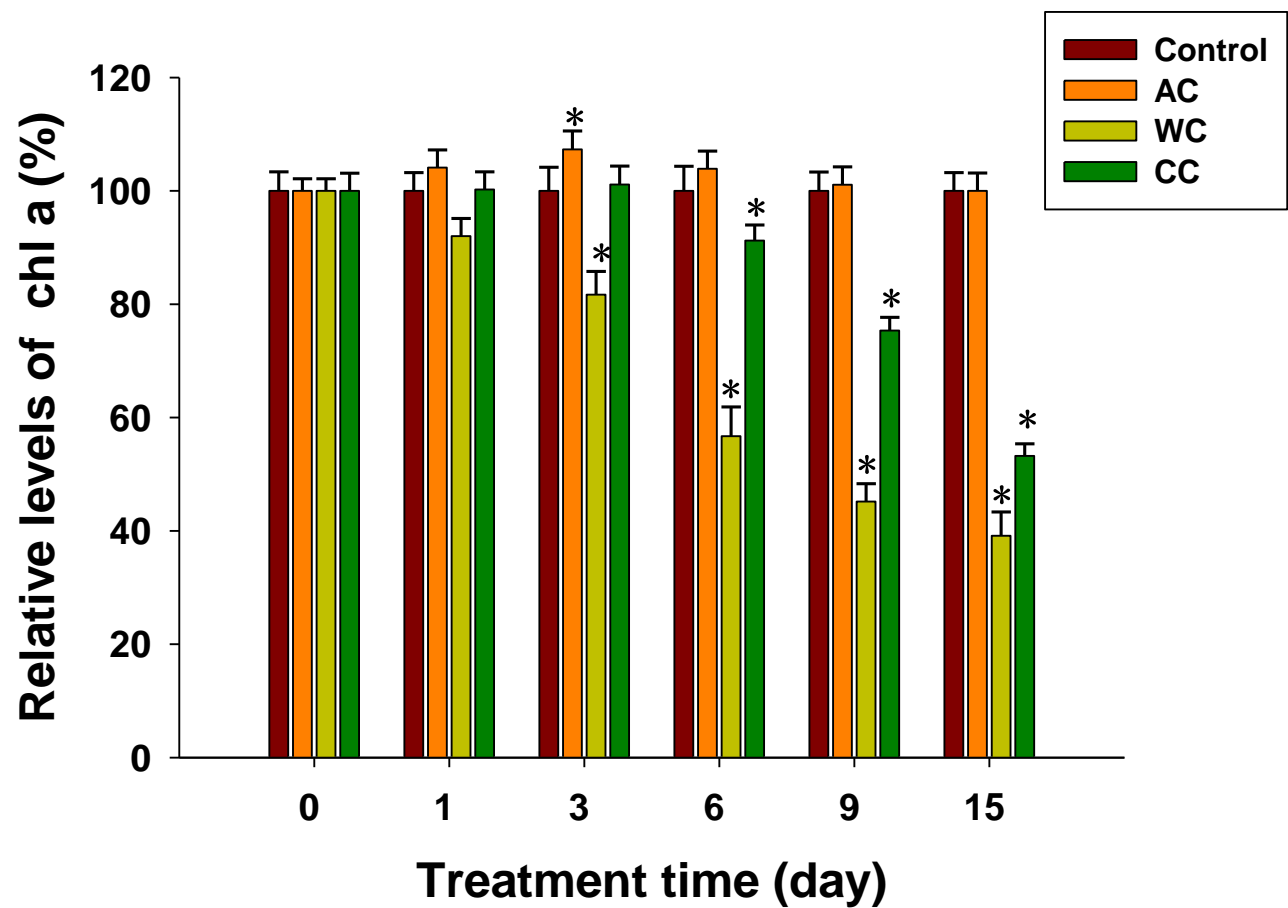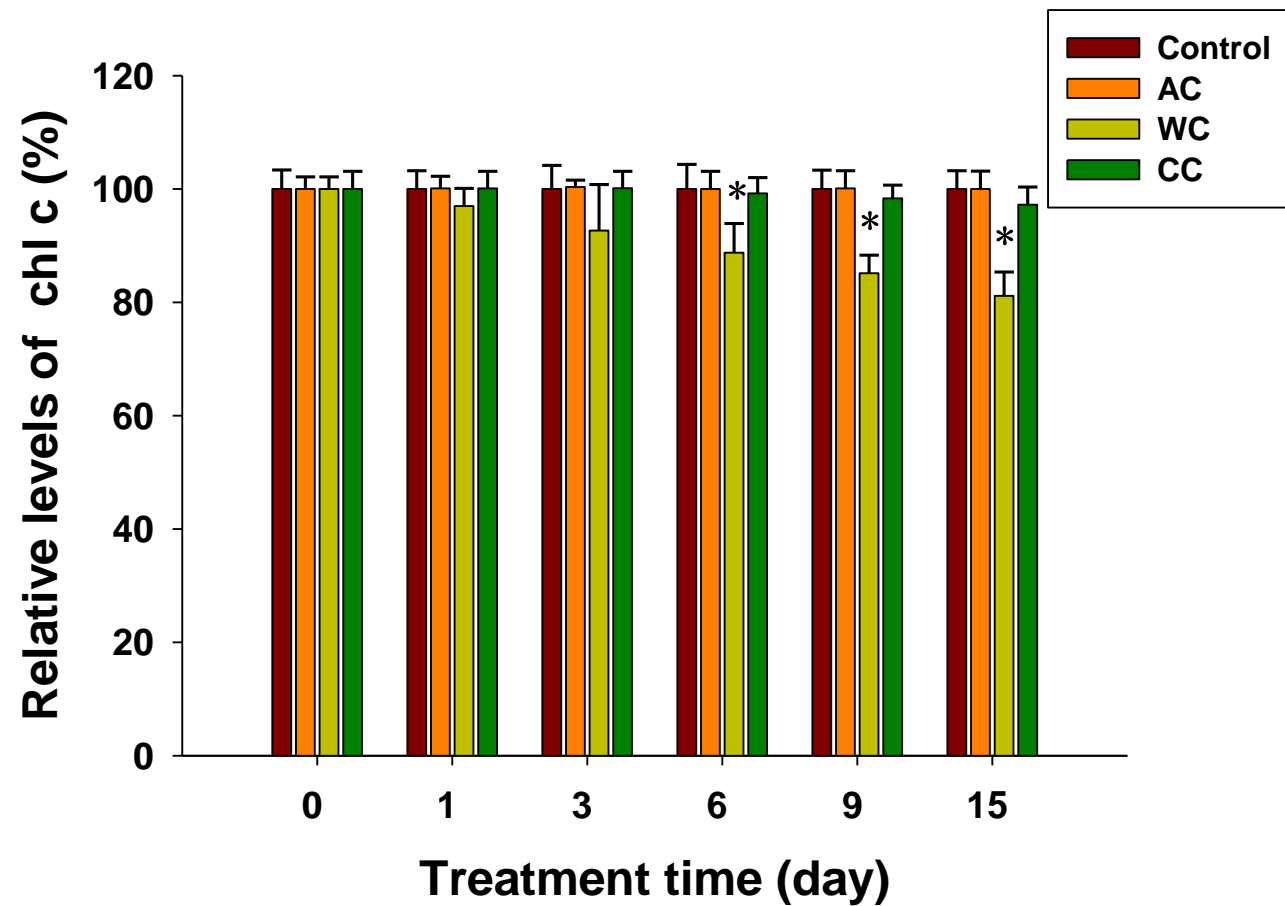

Supplement: Supplementary file 1 [file animals-12-02259-s001.zip › Supplementary Materials/Supplementary Figures/Figure S3 .pdf]
